# Extracellular vesicle tissue factor and tissue factor pathway inhibitor are independent discriminators of sepsis-induced coagulopathy

**DOI:** 10.1016/j.rpth.2024.102596

**Published:** 2024-10-18

**Authors:** Anna K. Tobiasch, Georg F. Lehner, Clemens Feistritzer, Andreas Peer, Birgit Zassler, Viktoria M. Neumair, Sebastian J. Klein, Michael Joannidis

**Affiliations:** 1 Division of Intensive Care and Emergency Medicine, Department of Internal Medicine, Medical University Innsbruck, Innsbruck, Austria; 2 Department of Internal Medicine V, Haematology and Oncology, Medical University of Innsbruck, Innsbruck, Austria; 3 Internal Medicine II, Gastroenterology, Hepatology and Rheumatology, Karl Landsteiner University of Health Sciences, University Hospital St. Pölten, St. Pölten, Austria

**Keywords:** disseminated intravascular coagulation, endothelial cells, extracellular vesicles, sepsis, tissue factor, tissue factor pathway inhibitor

## Abstract

**Background:**

Sepsis-induced disseminated intravascular coagulopathy (DIC) remains a challenging clinical entity associated with significant morbidity and mortality. Endothelial injury or activation and extracellular vesicles (EV) are postulated as important determinants of DIC.

**Objectives:**

The aim of this study was to test the discriminatory ability of E-selectin, EV, tissue factor (TF) and TF pathway inhibitor (TFPI) in sepsis-induced coagulopathy.

**Methods:**

In this prospective, single-center study, we collected plasma samples within 24 hours after sepsis diagnosis and followed these patients for 5 consecutive days. Overt DIC was determined by the International Society on Thrombosis and Haemostasis (ISTH) DIC score. Eighty-seven sepsis patients were recruited (35 with overt DIC) who presented with increased levels of EV, EV-associated TF procoagulant activity (TF-PCA), E-selectin, TF, and TFPI at admission compared with healthy subjects.

**Results:**

Only TFPI levels and TF-PCA discriminated between sepsis patients with or without DIC (area under the curve = 0.76; *P* = .0002). Increased TF-PCA was not sensitive in detecting sepsis-associated DIC; however, levels above 1.38 pg/mL showed high specificity in this cohort (sensitivity 27%, specificity 95%). The hazard ratio to progress to DIC over 5 days was 1.14 (95% CI, 0.64-2.07) for TF-PCA levels of 0.5 pg/mL or higher and 3.18 (95% CI, 1.74-5.79) for TFPI levels of 22.28 ng/mL or higher at admission.

**Conclusion:**

These findings highlight the pivotal roles of TF-PCA and TFPI in an early phase of sepsis-induced DIC. Only EV-associated and functionally active TF and not TF antigen levels showed a predictive potential regarding DIC. These novel results might support the improvement of diagnostic or even therapeutic strategies to mitigate the devastating consequences of DIC in septic patients.

## Essentials

• Endothelial activation might be relevant for DIC.
• We compared extracellular vesicles and endothelial markers in patients with or without DIC.
• High tissue factor pathway inhibitor levels at admission had the strongest association with DIC.
• This study highlights the role of the tissue factor pathway in sepsis-induced DIC.

## 1 Introduction

Sepsis is a severe and systemic inflammatory reaction to infection, characterized by a dysbalanced immune response [1]. Sepsis is an acutely life-threatening condition; however, its correct assessment remains a challenge, as the complex pathophysiology is extraordinarily heterogeneous. While adequate organ perfusion is an important determinant of organ dysfunctions, tissue perfusion in sepsis is jeopardized by multiple hemodynamically effective factors, including reduced cardiac function, vasodilation [2], and extravasation of fluid [1,3].

Alterations of the coagulation system are considered key mechanisms of the host response to infection and are frequently observed in sepsis patients (reviewed by Iba et al. [4]). The deterioration of this finely balanced system is a central pathophysiologic mechanism in the progression of sepsis severity, which can culminate in overt disseminated intravascular coagulopathy (DIC) [5].

There are 2 main mechanisms that are suggested as initiators of intravascular coagulation in inflammation. Exposure of the negatively charged phospholipid phosphatidylserine (PS) on cell surfaces is considered one key characteristic of the “procoagulant phenotype” acquired by different cells upon an inflammatory stimulus. It facilitates the formation of the prothrombinase complex, which enables the generation of thrombin [[6], [7], [8], [9]]. The second proposed pathway is via coagulation initiation by intravascular exposure of tissue factor (TF). While TF is expressed constitutively in virtually all somatic cells, its exposition on the cellular surface of blood cells is tightly regulated and inducible by a number of proinflammatory stimuli, including tumor necrosis factor α, interleukin (IL)-1β, or IL-6 [10]. Higher levels of intravascular TF were found to be associated with an increased risk for thrombosis in various diseases, especially in cancer patients and in patients with severe inflammatory response syndromes, such as sepsis [[11], [12], [13], [14]]. Both PS and TF are also intravascularly available on the surface of extracellular vesicles (EVs) [15,16]. EVs were shown to activate both prothrombin by PS exposure and factor (F)X by TF exposure, and this activity is increased in patients with sepsis [[17], [18], [19]]. As coexposure of PS increases TF activity [[20], [21], [22]], there might be a difference regarding the prognostic value between EV-associated TF and TF antigen concentrations in the pathogenesis of sepsis-associated coagulopathy.

TF pathway inhibitor (TFPI) is the predominant inhibitor of the coagulation initiation complex formed with TF. First, TFPI binds FXa [23]. This binary complex then binds to tissue factor-factor VIIa complex and forms a quaternary complex, abrogating all enzymatic activities of the included proteases [24]. While TFPI, in its soluble form, can inactivate FXa in the plasma, its main site of action is on the surface of endothelial cells (ECs) [25]. We recently showed a major role of the ratio between surface TF and TFPI on ECs in regulating net procoagulant activity on different lineages of ECs [26]. Notably, there is a large body of evidence showing a central role of TF activity by blood monocytes and monocyte-derived TF^+^ EVs in systemic inflammatory conditions such as endotoxemia (reviewed in Sachetto and Mackman [27]), highlighting the role of immune cells in immunothrombosis [28,29]. Evidence showing *in situ* TF expression by the endothelium, in contrast, is scarce [[30], [31], [32]]. *In vitro* experiments showed that ECs provide both a surface for coagulation initiation by expression of TF, coagulation amplification by exposition of PS, and consequent formation of the prothrombinase complex. Depending on signals conveyed by the blood or from the surrounding tissue, the endothelium is capable of either promoting anticoagulant or prothrombotic responses. The description of the angiopoietin-Tie2 axis, which was discovered in an unbiased proteome analysis of septic patients with vs without DIC, strongly underlined the active role of the endothelium in determining whether a sepsis patient progresses to DIC or not [33]. Endothelial activation or injury is therefore considered a hallmark of sepsis-induced coagulopathy (SIC) [34,35]. Soluble E-selectin is a marker of endothelial injury and was shown to correlate with the development of infection-associated DIC but not with other noninfectious underlying diseases [36,37]. We recently showed that E-selectin expression follows a dose-dependent increase in different EC lineages; therefore, it could reflect the degree of endothelial activation upon inflammatory stimuli *in vivo* [26].

With this study, we wanted to test whether levels of EVs, EV-associated and functionally active TF, and TF antigen or its counterpart TFPI correlate with E-selectin in a prospective cohort of sepsis patients with or without overt DIC in order to investigate whether a connection between endothelial activation and the manifestation of DIC can be substantiated. These parameters were further tested for their potential to discriminate sepsis patients with vs without overt DIC in a multivariable logistic regression model by following their trajectories over 5 consecutive days.

## 2 Methods

### 2.1 Study design and setting

This was a prospective observational monocenter study conducted at the medical intensive care unit (ICU) and intermediate ICU at the department of internal medicine of the Medical University Innsbruck. The active recruitment phase lasted from January 2017 to December 2019. The study was conducted in accordance with Austrian laws and regulations and the ethical principles regarding medical research involving human subjects formulated in the Declaration of Helsinki and was approved by the institutional ethics review board of the Medical University Innsbruck (study AN2013-0006). This study was funded by the Austrian National Bank fund (project number 15708) and the Austrian Society for Internal and General Intensive Care and Emergency Medicine (ÖGIAIN, project funding 2019).

### 2.2 Eligibility criteria

Adult patients (≥18 years), regardless of preexisting comorbidities, with clinically suspected or microbiologically proven infection, meeting the Surviving Sepsis Campaign 2008 criteria for sepsis or septic shock were eligible if the diagnosis of sepsis was within 24 hours before study inclusion [38,39]. To limit the influence of death as a competing risk, moribund patients with a life expectancy below 24 hours were excluded. Moreover, pregnant/breastfeeding subjects and those with cardiogenic shock, even if infection-related, were excluded from study participation. All included patients provided written informed consent. In addition to the defined eligibility criteria, all patients also fulfilled the Sepsis-3 criteria [1].

Healthy volunteers aged 18 or older who consented to study participation were included to form the control population. Subjects were not included if any medication interfering with platelet function or the plasmatic coagulation system was taken or when the subject was pregnant or breastfeeding.

### 2.3 Clinical data

To characterize the patient cohort, demographics, medical history, vital sign documentation, concomitant medication, and laboratory findings during the study period were collected. To compare the disease severity, the Sequential Organ Failure Assessment Score [40] and the Simplified Acute Physiology Score II [41] were calculated for each patient. To test the primary endpoint, patients were grouped into patients with overt DIC, defined by an ISTH-DIC score of greater or equal to 5 points, including the parameters prothrombin time, fibrinogen, platelet count, and D-dimer, and patients with no overt DIC, ie, scoring less than 5 points [42]. In addition, the score for SIC suggested by Iba et al. [43], which contains the items platelet count, INR, and Sequential Organ Failure Assessment score, was calculated. Septic shock was defined as sepsis-associated initiation of vasopressor treatment. In addition to the primary endpoint, DIC and the survival of patients after 7 and 28 days were documented.

### 2.4 Specimen collection

Citrated blood samples of critically ill patients were drawn from an arterial line. Citrated blood samples from healthy volunteers were collected by venepuncture of the antecubital vein. In both settings, the first 5 mL of blood were discarded. Platelet-poor plasma was obtained after centrifugation at 1500 × *g* for 15 minutes. Platelet-poor plasma obtained from the first centrifugation step was spun for an additional 2 minutes at 13,000 × *g* to obtain platelet-free plasma. Supernatants were stored at −80 °C until applied to the *in vitro* tests for a maximum of 12 months. For *in vitro* assays, 3 consecutive patients with and without sepsis-induced DIC were selected. All patient data were anonymized for analysis.

### 2.5 Laboratory data

Laboratory data were collected for 5 consecutive days. In addition to parameters assessed as part of daily care at the central laboratory of the Medical University Innsbruck, which included values to evaluate infection and inflammation, coagulation, and organ dysfunction (see Supplementary Table S1 and Supplementary Methods), additional parameters specific to the research question were measured by the experimenters.

EVs were enriched by the sequential centrifugation method with a maximal speed of 20,000 × *g* from citrated plasma samples, as described in Supplementary Methods [44]. EV concentration was determined using a commercial assay (Zymuphen MP Activity Kit, Hyphen BioMed), which detects coagulation active PS equivalents. Functionally active TF on the surface of EV preparations was measured by a chromogenic FX activation assay (Zymuphen MP-TF Activity Kit, Hyphen BioMed). Levels of E-selectin (CD62E) were determined in EV-depleted citrated plasma samples of patients and healthy controls with a commercial immunoassay (E-selectin Soluble Human ELISA Kit, Invitrogen); levels of TFPI expressed by the TFPI1 gene were detected with Human TFPI Immunoassay (Quantikine Elisa Kit, DTFP10, R&D Systems), which recognizes regions of the N-terminus of TFPI; total TF antigen concentration was determined with IMUBIND TF-ELISA (Sekisui Diagnostics LLC) according to the manufacturer’s manual.

### 2.6 Statistical analysis

Statistical analyses were performed with R (R Foundation for Statistical Computing, version 4.0.3), RStudio (version 1.1.463, Posit), and GraphPad Prism (version 9.0.2). Outcome figures were produced with GraphPad Prism, R, and Inkscape (version 1.0 beta1, www.inkscape.org).

Descriptive statistics included means with SDs, median with IQR, and frequency data for continuous and categorical variables. Differences between 2 or more groups were tested by *t-*test and 1-way analysis of variance or nonparametric alternatives as indicated.

To test the discriminative ability of parameters, an unconditioned binary logistic regression model was developed. Only complete sepsis cases were included; missing cases were considered to have occurred at random. The dependent variable represented the fulfillment of ISTH-DIC criteria at study inclusion. The α-error level for model retention was set at *P* ≤ .05. A stepwise-backward process, based on the study hypothesis, was employed for model fitting. Wald's test, Akaike Information Criterion, and the area under the curve (AUC) were used for model comparisons and relative fit assessment. The best-fitting model was then used to evaluate predictors for DIC development in sepsis, interpreting odds ratios with 95% CIs derived from model coefficients and SEs.

To calculate the probability of developing overt DIC over the observational period, a Kaplan–Meier survival analysis was performed, and log-rank hazard ratios (HRs) were derived to compare the curves between groups. Survival curves were plotted to visualize the probability of developing DIC over time. Each participant’s “survival time” was defined as the time point when a patient developed overt DIC. Differences between curves were tested with a log-rank (Mantel–Cox) test, and HRs (log-rank) are reported as ratios and corresponding 95% CIs. The α-error level was set at ≤.05 for all tests.

## 3 Results

### 3.1 Descriptive analysis

**Figure 1:**
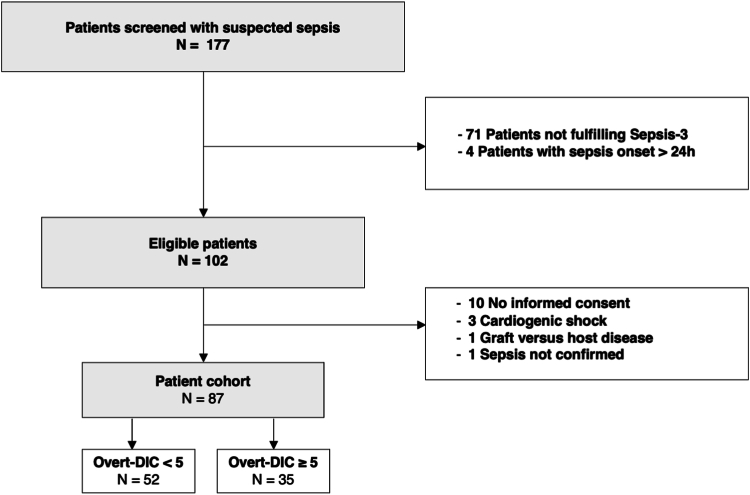
Flow chart of patient screening and study inclusion. DIC, disseminated intravascular coagulopathy.

Overall, 177 patients with suspected sepsis were screened after admittance to the medical ICU between January 2017 and December 2019. Seventy-five patients did not meet inclusion and exclusion criteria; as such, 102 patients were eligible. No retrospective informed consent was obtainable from 10 included patients; further, 3 patients were retrospectively excluded as the primary diagnosis was adapted after admission to the ICU, and 2 subjects were excluded from the analysis due to cardiopulmonary resuscitation following cardiac arrest before the first blood sampling. In total, 87 septic patients and 16 healthy volunteers were included in the final analysis set (Figure 1).

Table 1 summarizes demographic and clinical data; 86 patients were admitted for medical reasons and 1 postsurgical patient, with common comorbidities being oncologic disease (25%) and hepatic cirrhosis (20%). The mean age was 61 years; 44% were female. At study inclusion, 40.2% of patients reached an ISTH overt DIC score of ≥5, with 29% women and 49% men (*P* = .095). Over 5 days, 11 more patients met ISTH-DIC criteria. Septic shock occurred in 75.9% of patients at ICU admission, and was more prevalent in men than in women (63% vs 86%; *P* = .03).

**Table 1:** Clinical characteristics of the study cohort.

Parameter	Total *N = 87*	DIC *n = 35*	No DIC *n* = 52
Age (y)	63 (53-73)	58 (50-70)	64 (55-75)
Sex, female	38 (44)	11 (31)	27 (52)
Race			
Caucasian	86 (99)	35 (100)	51 (98)
Black	1 (1)	0 (0)	1 (2)
SIC at admission	56 (64)	35 (100)	21 (40)
ISTH overt DIC score	4 (3-5)	5 (5-6)	3 (2-4)
SIC score	4 (3-5)	5 (5-6)	3 (2-4)
SOFA score	12 (7-14)	13 (12-15)	9 (6-12)
SAPS II score	52 (39-66)	64 (42-75)	50 (37-63)
7-d mortality	15 (17)	13 (37)	2 (4)
28-d mortality	25 (29)	17 (49)	8 (15)
Septic shock	66 (76)	30 (86)	36 (69)
Focus of infection			
Pneumonia	37 (43)	13 (37)	24 (46)
UTI	12 (14)	2 (6)	10 (19)
Multiple foci	12 (14)	8 (23)	4 (8)
Bacteremia	55 (63)	26 (74)	29 (56)
Gram-negative	33 (38)	18 (51)	15 (29)
Gram-positive	22 (25)	8 (23)	14 (27)
Fungaemia	8 (9)	4 (11)	4 (8)
Systemic candidiasis	4 (5)	2 (6)	2 (4)
Invasive aspergillosis	6 (7)	2 (6)	4 (8)
Viremia	14 (16)	6 (17)	8 (15)
CMV	5 (6)	2 (6)	3 (6)
Influenza A/B	3 (3)	1 (3)	2 (4)
Cirrhosis hepatitis	8 (9)	5 (14)	3 (6)
Chronic kidney disease	16 (18)	5 (14)	11 (21)
AKI at admission	44 (51)	21 (60)	23 (44)
Renal-replacement therapy	28 (32)	15 (43)	13 (25)
Mechanical ventilation^a^	37 (43)	17 (49)	20 (38)
Haemato-oncologic disease	20 (23)	11 (31)	9 (17)
AML	7 (8)	7 (20)	0 (0)
ALL	5 (6)	4 (11)	1 (2)
Subacute	8 (9)	0 (0)	8 (15)
Status posttransplantation	12 (14)	5 (14)	7 (13)
Liver transplantation	6 (7)	2 (6)	4 (8)

Displayed are the median with IQR and *n* (%).

AKI, acute kidney injury; ALL, acute lymphoytic leukemia; AML, acute myeloid leukemia; CMV, cytomegalia virus; DIC, disseminated intravascular coagulopathy; ISTH, International Society on Thrombosis and Haemostasis; SAPS, Simplified Acute Physiology Score; SIC, sepsis-induced coagulopathy; SOFA, Sequential Organ Failure Assessment; UTI, urinary tract infection.

a Includes patients with non-invasive ventilation and intubated patients.

DIC patients had higher disease severity scores, a higher risk of death within 28 days (relative risk [RR], 1.9; *P* = .01), and a greater risk within the first 7 days (RR, 4.5; *P* = .001). The most common infection focus for both groups was the lung. DIC patients had more confirmed infection foci, with 51% having Gram-negative bacteria (vs 29% in septic patients without DIC). The most common comorbidity was hematologic malignancies (23%), with 11 of 20 hemato-oncologic patients reaching a DIC score of ≥5. Twelve patients developed sepsis under immunosuppression after transplantation and 6 after liver transplantation. However, prior liver transplantation did not increase the DIC risk in this cohort (RR, 0.8; *P* = .32).

At admission, C-reactive protein levels did not differ significantly between septic patients with or without DIC. However, procalcitonin and IL-6 levels were significantly higher in DIC patients. While tumor necrosis factor α levels were mostly below the detection limit, DIC patients appeared to have higher levels. DIC patients had significantly lower white blood cell and platelet levels. Creatinine and troponin T tended to be higher in DIC patients, though not statistically significant. Lactate levels were significantly higher in septic patients with an ISTH overt DIC score of ≥5, as were levels for N-terminal pro–B-type natriuretic peptide. These results are available in Supplementary Table S2.

In this study, the SIC score found 21 additional patients to be at risk for SIC. All patients who scored 5 or more points by the ISTH criteria also scored 4 or more points with the SIC system. While the SIC score can total a maximum of 6 points, compared with a maximum of 10 points for the ISTH overt DIC score, the median scores overlapped with a median of 4 points (IQR, 3-5) in both groups.

Although both thrombotic and bleeding events had a trend to a higher frequency in the DIC group, the differences were not statistically significant. The RR for a thrombotic incident slightly increased in the DIC group (RR, 1.2; *P* = .52), as did the risk for bleeding (RR, 1.3; *P* = .35). In our cohort, two-thirds of all patients (66%) were anticoagulated with at least 1 agent. Of these, 53% received low-molecular-weight heparin subcutaneously. Three patients were on direct oral anticoagulants, and only 1 patient had a FXa inhibitor as premedication. Seven patients (8%) received acetylsalicylic acid.

### 3.2 Coagulation and endothelial parameter concentrations at sepsis diagnosis

Categorizing the recruited sepsis patients by overt DIC vs no DIC revealed significantly higher D-dimer (4241 μg/mL; IQR, 2005-9003 μg/mL), prolonged activated partial thromboplastin time (43 seconds; IQR, 36-55 seconds), and a lower efficiency of prothrombin time (67%; IQR, 52%-83%) in DIC patients. Only 4 sepsis patients had fibrinogen levels below 150 mg/dL (2 patients with acute myeloid leukemia and 2 with hepatic cirrhosis), and all of those had DIC scores of ≥6 points. Antithrombin was significantly lower in patients with DIC (Table 2).

**Table 2:** Laboratory measurements of parameters of the coagulation system. Results are corrected by the Benjamini–Hochberg procedure to control for false discovery rates.

Parameter	Total *n= 87*	DIC *n = 35*	No DIC *n* = 52	*P* value
D-dimer (μg/L)	4241 (2005-9003)	7927 (4520-13,052)	2339 (1406-4607)	<.001
aPTT (s)	43 (36-55)	54 (45-59)	37 (34-44)	<.001
PT (%)	67 (52-83)	53 (38-61)	74 (65-91)	<.001
INR	1.30 (1.10-1.50)	1.45 (1.30-1.88)	1.20 (1.10-1.30)	<.001
Fibrinogen (mg/dL)	570 (419-641)	485 (363-610)	574 (489-672)	.066
Platelets (G/L)	103 (51-188)	48 (14-81)	167 (103-258)	<.001
Antithrombin (%)	59 (49-71)	52 (42-60)	65 (53-76)	.004
EV (nM)	28.34 (14.12-50.14)	29.62 (16.26-49.96)	27.85 (11.78-48.82)	.63
TF-PCA (TF eq pg/mL)	0.47 (0.13-1.47)	0.69 (0.25-1.60)	0.28 (0.06-1.42)	.12
TF (pg/mL)	100 (73-131)	104 (70-136)	100 (75-123)	.69
TFPI (ng/mL)	28.74 (21.63-53.18)	32.58 (25.47-67.48)	25.40 (18.76-45.00)	.01
E-selectin (ng/mL)	163.2 (67.9-391.8)	131.8 (49.3-414.7)	177.8 (82.9-370.7)	.54

aPTT, activated partial thromboplastin time; DIC, disseminated intravascular coagulopathy; EV, extracellular vesicle; INR, international normalized ratio; PT, prothrombin time; TF, tissue factor; TF-PCA, tissue factor procoagulant activity; TFPI, tissue factor pathway inhibitor.

**Figure 2:**
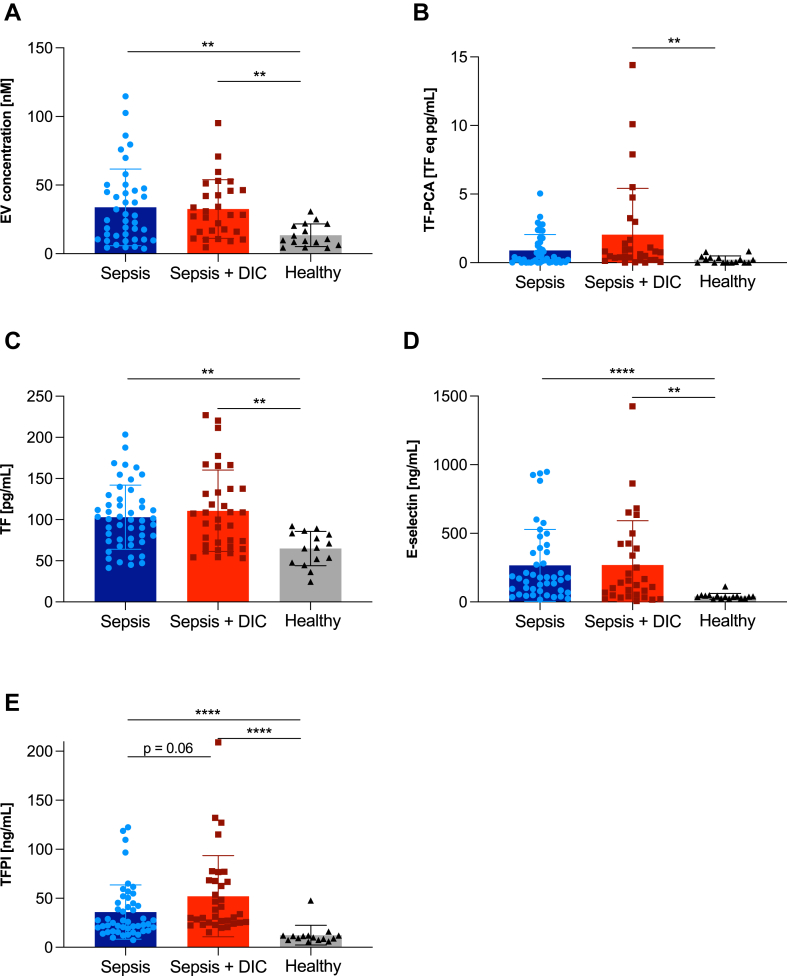
Comparison of extracellular vesicle (EV) concentration (A), tissue factor (TF) procoagulant activity (TF-PCA) on EVs (B), TF levels (C), E-selectin levels (D), and TF pathway inhibitor (TFPI) levels (E) in citrated plasma of healthy individuals (gray bars) and sepsis patients (Sepsis, blue bars, *n* = 52) and disseminated intravascular coagulopathy (DIC) patients, stratified by a DIC score of <5 or ≥5 (Sepsis + DIC, *n* = 35, red bars) were compared with levels measured in plasma of healthy volunteers (*n* = 16; A–E). Mean values with SDs are plotted. Differences between groups were tested with the Kruskal–Wallis Test followed by post hoc Dunn’s multiple comparisons test. ∗∗*P* < .01; ∗∗∗∗*P* < .0001.

As no reference ranges for the parameters EV concentration, TF procoagulant activity (TF-PCA), antigen levels, E-selectin, and TFPI concentrations were available, results were compared between sepsis patients and healthy volunteers. Median EVs, TF-PCA, immunologically measured TF levels, and E-selectin concentrations on the day of admission were higher in septic patients than in healthy volunteers. While post hoc tests confirmed a significant difference in both septic patients with and without DIC compared with healthy controls for EV concentration, TF, and E-selectin levels, TF-PCA was only significantly higher in septic patients with DIC (*P* = .003), while there was no significant difference between healthy volunteers and septic patients without DIC (*P* = .105). TFPI levels of sepsis patients were increased 2-fold at admission compared with baseline levels measured in samples from healthy volunteers (Figure 2).

Concentrations of EVs were higher in patients with DIC, as were functional TF activity on the surface of EVs; however, for both parameters, this difference was not statistically significant between septic patients with and without DIC. Levels of TF measured with an immunologic technique revealed an almost equal concentration in both groups. Conversely, soluble TFPI levels were significantly higher in patients with DIC (*P* = .01). In contrast, plasma levels of the endothelial injury marker E-selectin were higher in the non-DIC group.

### 3.3 Correlation analysis and predictive modeling of EV and endothelial parameters in sepsis-induced DIC

E-selectin only showed a weak but significant correlation with TF antigen levels (Spearman’s r = .31; 95% CI, 0.08-0.51; *P* = .008) but no significant associations with the other tested parameters. TF antigen levels also correlated significantly with TF-PCA (Spearman’s r = .43; 95% CI, 0.22-0.59; *P* < .001) and TFPI levels (Spearman’s r = .37; 95% CI, 0.16-0.55; *P* = .008). The strongest correlation was found between EV concentration and EV-associated TF-PCA (Spearman’s r = .54; 95% CI, 0.34-0.67; *P* < .001). Results are displayed in Supplementary Figure S1.

The start-up model imputes the variables of interest, defined by the hypothesis of the project and the results from the *in vitro* analysis. This unrestricted model thus contained EV concentration, EV-associated TF-PCA, plasma levels of TF antigen, TFPI, E-selectin, and the intercept of the model. Additionally, interactions between factors were computed and included in the follow-up model. The binomial link was defined as an ISTH overt DIC score ≥ 5 points. A *P* value of ≤.3 derived from z value comparisons was defined for variables to enter the next model. All measurements derived from healthy volunteers and included patients were analyzed (Supplementary Table S3).

Of 103 patients and controls, 87 had complete measurements for all variables. In the initial model, TFPI had the highest coefficient estimate, followed by TF-PCA. E-selectin showed a negative association with DIC. TF antigen and EV concentration had little contribution and were excluded. The only significant interaction (*P* = .25) was between EV concentration and TF levels, included in model 2. However, with an estimate of .19, the interaction had minimal contribution (*P* = .76) and was removed in model 3, resulting in improved efficiency without loss of performance. TFPI and TF-PCA significantly contributed to model 3, while E-selectin showed the lowest predictive performance. Model 4, with only TF-PCA and TFPI, showed no decrease in fit compared with model 3, achieving a moderate discriminatory ability (AUC, 0.76; *P* = .0002). Therefore, model 4 was considered the final prediction model (Table 3).

**Table 3:** Results of the multivariate logistic regression analysis.

Model	*Included factors*	Observations included	df	AIC	AUC	*P* value
1	EV, TF-PCA, E-selectin, TF, TFPI	87	81	105	0.7684	.0019
2	TF-PCA, E-selectin, TFPI, EV:TF	87	82	103	0.7696	.0008
3	TF-PCA, E-selectin, TFPI	87	83	101	0.7737	.0003
4	TF-PCA, TFPI	87	84	101	0.7573	.0002

AIC, Akaike Information Criterion; AUC, area under the curve; df, degrees of freedom of the calculated residual deviance; EV, extracellular vesicle; EV:TF, extracellular vesicle - tissue factor interaction; TF, tissue factor; TF-PCA, tissue factor procoagulant activity; TFPI, tissue factor pathway inhibitor.

Although the AUC of 0.76 was considered good, the generalized additive model indicated that only 15.7% of the variation could be explained by TFPI and TF-PCA. Given the multifactorial pathogenesis and diverse anamnestic factors, this performance was acceptable. The model's sensitivity was 84%, and specificity was 37%, with a positive predictive value (PPV) of 28% and a negative predictive value of 45%. The Youden index identified optimal cutoff values as follows: TFPI at 22.28 ng/mL (sensitivity 91%, specificity 56%) and TF-PCA at 1.38 pg/mL (sensitivity 27%, specificity 95%). The closest balance between sensitivity (62%) and specificity (58%) was found at 0.5 pg/mL for TF-PCA, though particularly low levels of TF-PCA caused ties in the modeling process.

High TFPI values exhibited high sensitivity, while elevated TF-PCA levels demonstrated high specificity in detecting DIC patients. The ratio of TF to TFPI on the endothelial membrane appeared crucial for net procoagulant activity. However, testing the TF-PCA/TFPI ratio for additional predictive potential did not yield significant results despite the individual good performance of TFPI and TF-PCA in the multiple logistic regression analysis (Supplementary Figure S2).

### 3.4 TF-PCA and TFPI: risk for sepsis-induced DIC over 5 consecutive days

**Figure 3:**
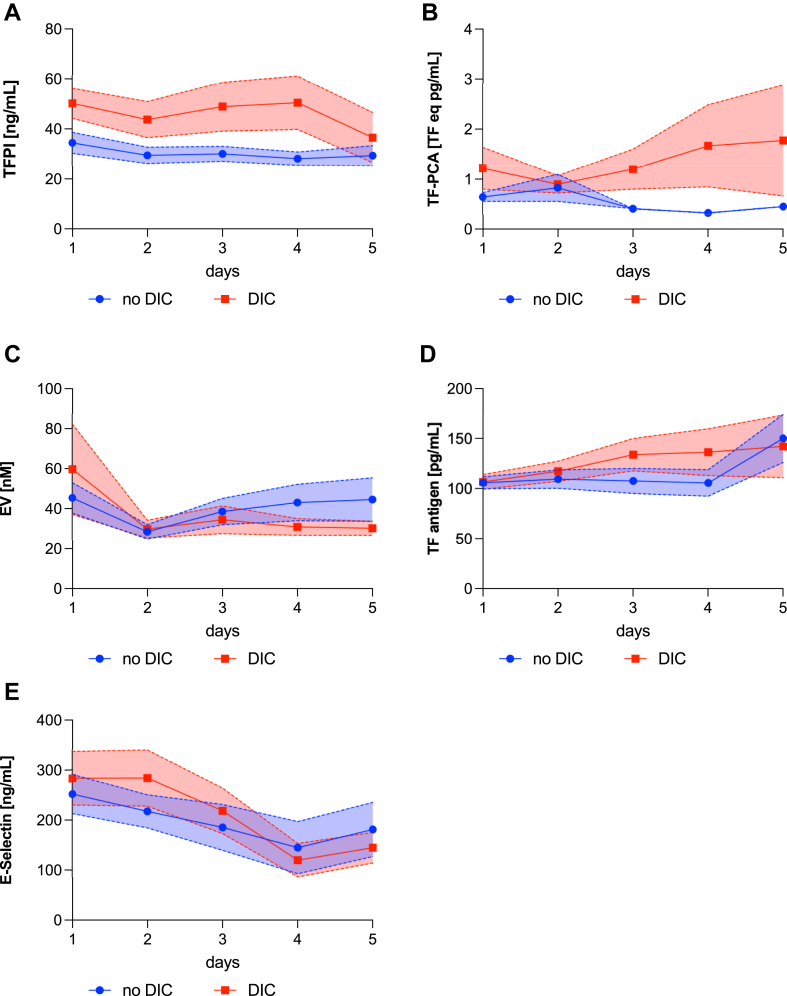
Development of tissue factor (TF) pathway inhibitor (TFPI) levels (A), TF procoagulant activity (TF-PCA, B), extracellular vesicle (EV) concentration (C), TF antigen (D), and E-selectin levels (E) measured in plasma samples from sepsis patients for 5 consecutive days following sepsis diagnosis. The blue line displays the trajectory of the 2 parameters in patients who never developed overt disseminated intravascular coagulopathy (DIC) vs patients who reached 5 or more points of the International Society on Thrombosis and Haemostasis DIC score. Lines indicate mean values with the corresponding SE.

To explore the dynamics of different parameters in our cohort, patients were observed for 5 consecutive days following the diagnosis of sepsis. The study cohort was divided into patients who had reached an ISTH-DIC score of 5 points or more (DIC) vs no overt DIC, and the trajectories of investigated parameters were plotted over time. TFPI levels, which were significantly higher on the day of admission, remained constantly higher in patients with DIC, except for measurements on day 5 after admission (Figure 3A). Levels of TF-PCA were marginally different on the day of admission but notably diverged from patients who did not develop overt DIC, showing a discrete increase over the time course (Figure 3B). The concentration of total EV (Figure 3C) was higher on the day of admission for DIC patients, but levels overlapped on the following days. While TF antigen levels (Figure 3D) were not different between groups on the day of admission, levels also did not change over time. In contrast, E-selectin (Figure 3E) decreased remarkably over the first 5 days after sepsis diagnosis in both DIC and non-DIC groups.

**Figure 4:**
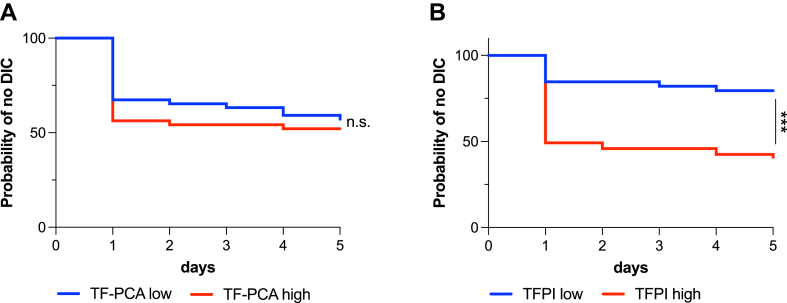
Kaplan–Meier curves displaying the probability of patients with high (red lines) or low levels (blue lines) of tissue factor procoagulant activity (TF-PCA, A) and tissue factor pathway inhibitor (TFPI, B) to develop overt disseminated intravascular coagulopathy (DIC). n.s., not statistically significant; ∗∗∗*P* < .001.

To further assess the utility of the determined cutoff values from the logistic regression model, we employed Kaplan–Meier survival analysis to calculate the probability of developing DIC during the observational period. Log-rank HRs were derived by dividing the study population into 2 groups: low or high TFPI levels at admission (Youden index cutoff: 22.28 ng/mL) and low or high TF-PCA levels at admission (cutoff: 0.5 pg/mL). No significant differences in DIC onset rates were observed between groups with high or low TF-PCA levels at admission (Mantel–Cox chi-square = 0.31; *P* = .58; Figure 4A). In contrast, significant differences were noted for TFPI levels (Mantel–Cox chi-square = 14.26; *P* < .0001; Figure 4B).

Consequently, the HR of developing DIC over 5 days with a TF-PCA level of 0.5 pg/mL or higher at admission was low (HR, 1.14; 95% CI, 0.64-2.07). However, TFPI levels of 22.28 ng/mL or higher suggested an increased risk (HR, 3.18; 95% CI, 1.74-5.79).

## 4 Discussion

This was a single-center prospective study combined with preclinical laboratory analyses aimed to explore the role of EVs and markers of endothelial activation in patients with sepsis. The importance of E-selectin, EVs, TF, functional TF activity, and its counterpart TFPI was assessed in this prospective cohort study with sepsis patients, and TF-PCA and TFPI were identified as relevant factors.

EV-associated TF-PCA and TFPI levels were identified as significant discriminators of sepsis patients with overt DIC vs those without overt DIC. Especially TFPI levels were significantly elevated in sepsis patients who also scored for overt DIC at admission. In contrast, except for a weak correlation with immunologically measured TF concentrations, levels of EVs, EV-associated TF-PCA, and TFPI showed no association with the degree of endothelial activation in sepsis, as reflected by plasma E-selectin levels.

The measured absolute levels of EV-TF-PCA are comparable with those reported in other published studies that investigated TF-PCA in the context of inflammation-induced coagulopathy. In a study published in 2020 by Schmedes et al. [45], a peak EV-associated TF-PCA of 0.51 pg/mL predicted intravascular coagulopathy with an AUC of 0.634 in patients with orthohantavirus infection. However, others have found higher concentrations, especially if in-house methods were used [46]. The analysis of data for the presented project included the subtraction of baseline values of blank control samples before the interpolation was performed. TF concentrations are low in human plasma samples and should not be detectable in the blood of healthy volunteers; therefore, the reported concentrations here were considered plausible, as others have found similar concentrations in plasma from septic patients [46,47]. However, the actual concentrations might have been underestimated in comparison with other more sensitive EV-TF-PCA measurement techniques [48]. In addition to different subtypes of TF, ie, soluble or membrane-bound, encrypted or decrypted, and its bioavailability to effectively interact with the coagulation system by appropriate available phospholipids, there are substantial limitations regarding the reproducibility of TF antigen measurements with different antigen assays displaying variable sensitivity and concordance with TF activity assays [49,50].

While reduced availability of endothelial surface TFPI together with an increase in TF expression resulted in increased procoagulant activity in cultured ECs *in vitro* [26], our study showed that elevated TFPI levels were the most relevant characteristic to distinguish DIC vs non-DIC patients in the multivariate logistic regression model. Increasing levels of TFPI, along with increasing levels of EV-associated TF-PCA, were found to go along with a higher risk for DIC.

The finding that TFPI plasma levels were higher in patients with more severe coagulopathy was unexpected, as TFPI is considered to diminish the activation of coagulation. In contrast, this finding underlines the complicated mechanisms related to the progression of coagulopathy in septic patients: inflammation is a potent driver of endothelial activation, as evidenced by the induction of E-selectin expression upon proinflammatory stimulation *in vitro* [26] and significantly higher levels in the plasma of sepsis patients in this study. While the gene expression of TF and E-selectin are strongly regulated in ECs by inflammation, TFPI showed no significant response, as found by gene set enrichment analysis of datasets across different EC subtypes and different applied proinflammatory stimulants [26]. While the expression of TFPI is not upregulated by inflammatory stimuli, shedding of TFPI from cellular surfaces was shown to occur in systemic inflammatory syndromes. For example, in a mouse model of endotoxemia, the observed decline in lung-associated TFPI activity was prevented in plasminogen knockout mice [51]. Follow-up experiments revealed a central role of plasmin in the shedding of TFPI from lung ECs, which also contributed to thrombotic complications [[51], [52], [53]]. The active shedding of TFPI from EC surfaces by enzymes such as plasmin and neutrophil elastase has also been observed in human inflammatory conditions, including sepsis and acute lung injury [51,52,54]. Therefore, there might be a connection between leukocyte, or especially neutrophil activation, and plasma TFPI levels.

Results of TFPI levels in sepsis patients are contradictory; while TF antigen levels were found to be an independent predictor of 30-day mortality in sepsis patients [55], this study did not detect significant differences in TFPI levels between healthy controls, patients with sepsis, and severe sepsis. More recently, a study investigating coagulation-related parameters in 100 patients with moderate COVID-19 disease at admission was found to have significantly higher plasma levels of TFPI compared with healthy control subjects [56]. Other groups also reported higher levels of TFPI before the onset of DIC compared with non-DIC patients, which peaked on the day of DIC onset [57]. Moreover, higher levels of TFPI were found to be associated with an increased risk for venous thromboembolism and all-cause mortality in 898 cancer patients. In accordance with our study, Englisch et al. [58] did not detect a correlation between TFPI and EV-associated TF either. Of interest, this study found a mediating or confounding effect of P-selectin levels on the predictive power of TFPI, which suggests that activated platelets might considerably contribute to the levels of intravascular TFPI. In the study presented here, levels of TFPI remained consistently higher within the first 4 days but were comparable with levels measured on day 5 with sepsis patients without overt DIC. TF-PCA levels, in contrast, continued to rise in most patients with DIC on the day of admission, while there was no dynamic in patients without overt DIC observable. This finding is contrasted by a recent study with a similar setup, which showed declining levels of EV-associated TF-PCA in septic DIC patients, as measured with an in-house assay to determine TF-mediated FX activation of enriched EV suspensions [47]. Notably, our study shows a great variance in the detected levels of EV-associated TF-PCA on day 5; therefore, a different outcome in other study cohorts is likely. More robust trajectories were detected for TFPI; higher levels of TFPI at admission suggest a lower probability of developing overt DIC over 5 consecutive days; however, progression rates beyond the first day were generally low in our cohort.

In this study, TF-PCA had a remarkably better performance compared with TF antigen levels. A determinant of TF functionality is its association with a phospholipid bilayer, and the release of TF on the surface of the EVs might be of higher functional relevance. This biological connection to TF-PCA was also evident in the correlation analysis, where EV concentration in plasma samples showed a significant correlation with functional activity of EV-associated TF. However, the contribution of EV concentration to predict the likelihood of DIC was low, and no significant interaction between EVs and TF-PCA was detected. This indicates that the EV concentration is not the defining factor of clinically relevant coagulation activation but rather the TF-bearing subpopulation of EVs.

A limitation common to all DIC-related research is the lack of a gold standard for DIC diagnosis. In this project, the diagnosis DIC is based on the ISTH-DIC score, which is composed of laboratory parameters. A Danish population-based study found a PPV of the ISTH-DIC score in patients with an overt DIC score ≥ 5 of 47% (95% CI, 35%-57%) and assessed the diagnosis of DIC on the basis of laboratory data and clinical signs or microthrombosis and/or bleeding according to a panel of physicians [59]. In this Danish study, the PPV increased to 88% for patients with an ISTH-DIC score of ≤7; the study results undermined that the score is susceptible to misinterpreted changes in laboratory values due to a different underlying disease, such as hematologic malignancies or liver diseases, and the source population considerably influences the accuracy of the score [59]. Notably, the published scores for the DIC assessment used trivial cutoff values, which were chosen to increase the simplicity of the application. The thresholds were defined by a statistical approach; therefore, the continuous nature of the predictor variables is included in the definition of the cutoff values. The SIC scoring system focuses on the detection of patients at risk of developing symptomatic DIC. Therefore, many patients with a positive SIC score still have a compensated form of DIC but are at higher risk of proceeding to overt DIC, which is considered as the decompensated sequel [60].

DIC is a common complication of sepsis; however, patients who develop severe sepsis frequently suffer from multiple serious comorbidities, and the contribution of coagulopathy to sepsis-related mortality probably varies depending on the patient’s underlying disease [61]. We included septic patients without further definition of underlying disease or focus, which led to a heterogeneous set of patients. While this limits an overall comparison of included patients, this approach reflects the patient population that is met by physicians and researchers in medical ICUs. In addition to its unique composition regarding cohort characteristics, the size of the cohort is rather small, so the generalizability to other ICU patients is limited. The exclusion of patients without informed consent and the lack of matching between healthy controls and sepsis patients could introduce bias in the study. Despite the wide range of underlying diseases, which included patients with an underlying hematologic condition and patients with advanced stages of chronic liver disease, both of which are particularly important when considering the endothelial contribution to DIC [59], the overall risk of mortality was found higher in patients with overt DIC. In hematologic patients with DIC, endothelial dysfunction is considered to be less significant compared with trauma patients or immunocompetent sepsis patients [60]; more severe thrombocytopenia, however, most probably has different causes compared with other patients with different comorbidities (reviewed by Iba et al. [61]). While the detection of a significant contribution of individual parameters to identify patients with DIC is remarkable in this heterogenous cohort, a detailed subgroup analysis would be of great interest; however, this study does not provide enough granularity and statistical power to allow for a meaningful deeper investigation. As mentioned above, pivotal scores used for statistical evaluation, like the ISTH overt DIC score, are biased to higher levels in hematologic malignancies, further complicating any analysis. Nonetheless, analysis of endothelial parameters indicates some contribution, as levels of circulating TFPI were not significantly different between patients with or without hemato-oncologic disease.

In conclusion, elevated levels of EV-associated TF-PCA and TFPI show a potential to identify patients with overt DIC independently of other classic coagulation parameters. The connection between biomarker levels and disease severity underscores their clinical relevance and prognostic value in the context of sepsis. Furthermore, the presented results suggest that higher levels of TF-PCA and TFPI already at admission are associated with a higher risk of developing DIC in septic patients. Plasma TFPI showed the strongest potential as an indicator and predictor of overt DIC in septic patients and could indicate alterations on the level of ECs.
