# Supplementary material for: Extracellular vesicle tissue factor and tissue factor pathway inhibitor are independent discriminators of sepsis-induced coagulopathy
Source: Res Pract Thromb Haemost. 2024 Oct 18;8(7):102596. doi: 10.1016/j.rpth.2024.102596 (PMC11609531; doi:10.1016/j.rpth.2024.102596)
Supplement: Supplemental Methods [file mmc1.docx]

**Supplementary to: Extracellular vesicle tissue factor and tissue factor pathway inhibitor are independent discriminators of sepsis-induced coagulopathy**

Anna K Tobiasch, Georg F Lehner, Clemens Feistritzer, Andreas Peer, Andreas Pircher, Birgit Zassler, Viktoria Haller, Sebastian J Klein, Michael Joannidis

# Supplementary Methods

## Enrichment of EV

Sampling and enrichment procedures were designed according to the current methodological guidelines for EV research [232]. The anticoagulant chosen was sodium citrate, samples of patients were obtained from an arterial line, whereas venous blood samples were obtained from healthy controls using a 21 gauge needle and minimal use of tourniquet. All individuals included into the study were non-fasted.

Enrichment of EV was started maximal 30 minutes after blood sample collection with a differential centrifugation protocol: citrated plasma was centrifuged for 15 minutes at 1500 x g with slow deceleration to obtain platelet-poor plasma. After transferring the platelet poor plasma into fresh plastic tubes, the samples were again spun for 2 minutes at 13000 x g again at room temperature. This step pellets platelets and large apoptic bodies. The obtained supernatant or platelet free plasma (PFP) was then again transferred into fresh 2 mL tubes and further subjected to two high-speed centrifugation steps for 30  minutes at 20000 x g and 4°C with a washing step with PBS-/- (without calcium or magnesium, Gibco, Invitrogen) in between to remove plasma residuals. EV pellets obtained from 1000 µL plasma were each resuspended in 150 µL sterile filtrated PBS-/-. Plasma samples with evident haemolysis were not used for EV isolation.

## Routine laboratory diagnostics

Coagulation parameters were measured on a BCS XP (Siemens Healthcare Diagnostics GmbH, Vienna, Austria): prothrombin time (Quick, Siemens Thromborel S), aPTT (Siemens Pathromtin SL), fibrinogen (Siemens Multifibren U), antithrombin (FIIa-based Siemens Berichrom Antithrombin III) and D-Dimer levels are provided in fibrinogen equivalent units (FEU), measured with Dade Innovance D-Dimer, Siemens).

C-reactive protein, procalcitonin (electro-chemi-luminescence method), B-type natriuretic pro-peptide (NT-pBNP), enzymatic creatinine and lactate-dehydrogenase were measured on a Cobas 8000 platform (Roche Diagnostics, Switzerland). Interleukin-6 was measured with BD OptEIA, human IL-6 ELISA set (BD Biosciences).

Lactate levels are derived from arterial blood gas measurements with the point-of-care diagnostic device (Radiometer ABL800 Flex, Radiometer GmbH, Germany). The highest levels measured with 24 hours were documented.

# Supplementary tables

**Supplementary table 1:**

Measurements assessed as part of routine laboratory diagnostics.

| Parameter | Specimen | Time point |
| --- | --- | --- |
| D-Dimer [µg/L] | citrated plasma | Day 1 – 5 |
| CRP [mg/dL] | heparinized plasma | Day 1 – 5 |
| PCT [µg/L] | heparinized plasma | Day 1 – 5 |
| IL-6 [pg/mL] | heparinized plasma | Day 1 – 5 |
| Platelets [G/L] | EDTA whole blood | Day 1 – 5 |
| WBC [G/L] | EDTA whole blood | Day 1 – 5 |
| Hematocrit [%] | EDTA whole blood | Day 1 – 5 |
| RDW (%) | EDTA whole blood | Day 1 – 5 |
| Creatinine [mg/dL] | heparinized plasma | Day 1 – 5 |
| LDH [U/L] | heparinized plasma | Day 1 – 5 |
| Lactate [mg/dL] | heparinized plasma | Day 1 – 5 |
| hsTroponin T [ng/L] | serum | Day 1 – 5 |
| NT-pBNP [ng/L] | heparinized plasma | Day 1 – 5 |
| aPTT [sec] | citrated plasma | Day 1 – 5 |
| PT [%] | citrated plasma | Day 1 – 5 |
| INR | calculated from PT (Quick) | Day 1 – 5 |
| Fibrinogen [mg/dL] | citrated plasma (Clauss) | Day 1 – 5 |
| Antithrombin [%] | citrated plasma | Day 1 – 5 |

**Supplementary table 2:**

Clinical chemistry measurements. LDH = lactate dehydrogenase;

|  | **Total**  *n = 87* | **DIC**  *n = 35* | **no DIC**  n = 52 | **p value** |
| --- | --- | --- | --- | --- |
| CRP [mg/dL] | *22.82 (16.55 - 32.41)* | *21.13 (14.27 - 31.16)* | *24.89 (16.79 - 32.56)* | *0.574* |
| PCT [µg/L] | *16.44 (4.28 - 58.87)* | *29.23 (7.99 - 68.29)* | *12.92 (2.63 - 56.12)* | *0.078* |
| IL-6 [pg/mL] | *213.5 (51.4 - 1999.5)* | *490.6 (165.2- 8418.3)* | *119.4 (31.4 - 499.8)* | *0.003* |
| WBC [G/L] | *12.1 (2.9 - 20.8)* | *8.0 (7.0 - 17.7)* | *14.7 (7.1 - 21.3)* | *0.043* |
| Hematocrit [%] | *29 (24.8 - 33.9)* | *27 (24 - 34)* | *31 (26 - 34)* | *0.205* |
| increased RDW (%) | *29 (33)* | *15 (43)* | *14 (27)* | *0.189* |
| Creatinine [mg/dL] | *1.80 (1.10 - 2.79)* | *2.16 (1.19 - 3.04)* | *1.76 (1.11 - 2.61)* | *0.391* |
| LDH [U/L] | *250 (179 - 387)* | *300 (203 - 540)* | *234 (169 - 305)* | *0.053* |
| Lactate [mg/dL] | *22 (15 - 39)* | *28 (19 - 52)* | *18 (11 - 32)* | *0.005* |
| hsTroponin T [ng/L] | *49 (22 - 102)* | *59 (30 - 80)* | *44 (18 - 107)* | *0.455* |
| NT-pBNP [ng/L] | *4588 (1551 - 14753)* | *8727 (2053 - 17695)* | *2646 (1129 - 7409)* | *0.020* |

**Supplementary table 3:**

Results of the logistic regression start-up model. Estimate = beta coefficient estimate; SE = standard error of estimate; z value = logistic regression coefficient normalized by standard error; p values were derived from z value comparisons.

|  | ***Included factors*** | **estimate** | **SE** | **z value** | **p value**  **(<\|z\|)** |
| --- | --- | --- | --- | --- | --- |
| Start-up Model | Intercept | *-12.00* | *4.01* | *-2.99* | *0.0028* |
|  | TFPI | *2.72* | *0.97* | *2.82* | *0.0048* |
|  | TF-PCA | *0.32* | *0.16* | *1.98* | *0.0478* |
|  | E-selectin | *-0.64* | *0.55* | *-1.16* | *0.2468* |
|  | TF | *0.26* | *1.66* | *0.16* | *0.8732* |
|  | EV concentration | *0.18* | *0.63* | *0.30* | *0.7646* |
|  |  |  |  |  |  |
| Interactions |  |  |  |  |  |
|  | EV:TF-PCA | *0.26* | *0.75* | *0.35* | *0.7297* |
|  | EV:E-selectin | *-0.93* | *1.49* | *-0.62* | *0.5345* |
|  | EV:TF | *6.01* | *5.23* | *1.15* | *0.2504* |
|  | EV:TFPI | *0.08* | *2.78* | *0.03* | *0.9773* |
|  | TF-PCA:E-selectin | *0.13* | *0.70* | *0.18* | *0.8579* |
|  | TF-PCA:TF | *0.29* | *1.30* | *0.23* | *0.8214* |
|  | TF-PCA:TFPI | *-0.90* | *1.19* | *-0.75* | *0.4507* |
|  | E-selectin:TF | *-0.03* | *3.67* | *-0.01* | *0.9936* |
|  | E-selectin:TFPI | *0.04* | *2.02* | *0.02* | *0.9844* |
|  | TF:TFPI | *-5.45* | *7.10* | *-0.77* | *0.4422* |

# Supplementary figures

**Supplementary figure 1:**


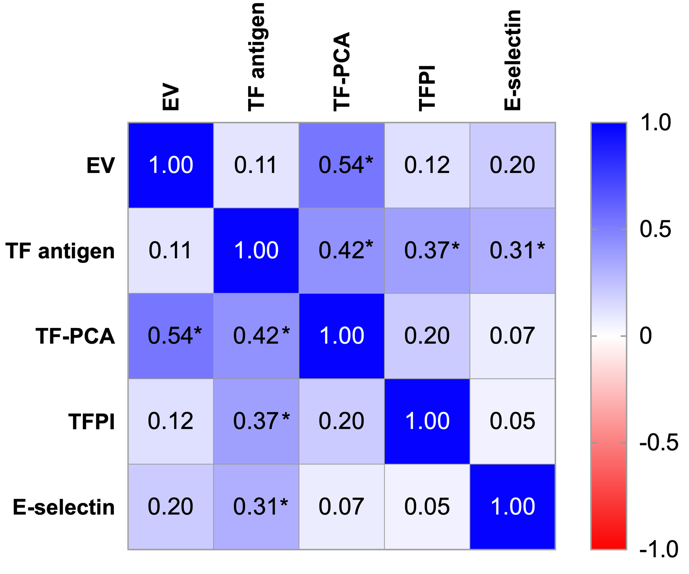


Supplementary figure 1: Correlation matrix of the variables of interest. A high positive Spearman’s r is color-coded in blue shades, a negative Spearman’s correlation coefficient in red. Correlations with a p of < 0.05 are marked with an asterisk.

**Supplementary figure 2:**


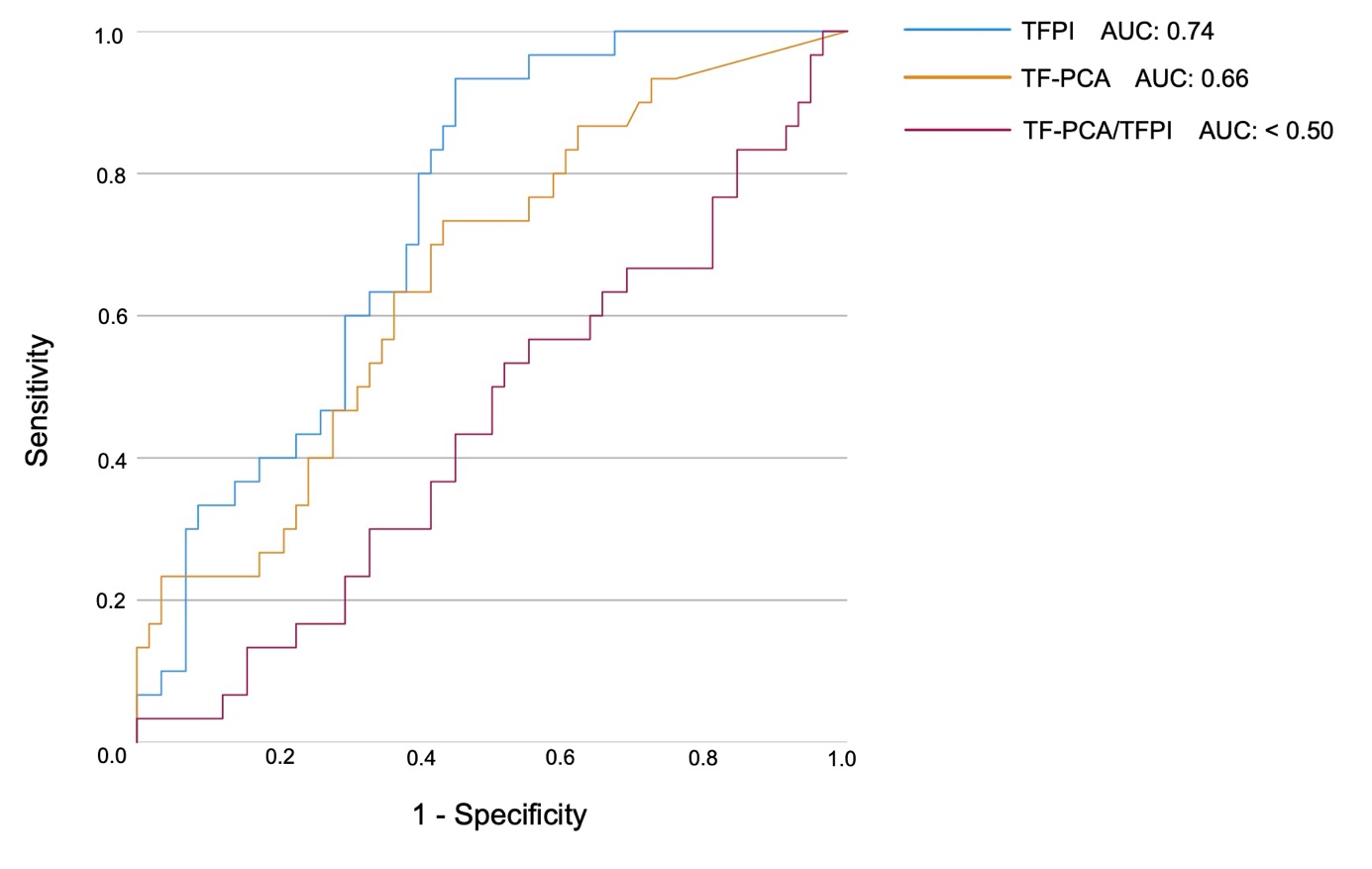


Supplementary figure 2: ROC curve comparing three different predictors of DIC, tissue factor pathway inhibitor (TFPI), tissue factor procoagulant activity (TF-PCA) and a ratio of of TF-PCA per TFPI. AUC = area under the curve.
